# Adaptation of short version of questionnaire for assessing the childbirth experience (QACE) to the Iranian culture

**DOI:** 10.1186/s12884-020-03317-9

**Published:** 2020-10-12

**Authors:** Mojgan Mirghafourvand, Mohammad Asghari Jafarabadi, Solmaz Ghanbari-Homayi

**Affiliations:** 1 Social determinants of Health Research Center, Tabriz University of Medical Sciences, Tabriz, Iran; 2 Department of Statistics and Epidemiology, Tabriz University of Medical Sciences, Tabriz, Iran; 3 Road Traffic lnjury Research Center, Tabriz University of Medical Sciences, Tabriz, Iran; 4 Department of Midwifery, Faculty of Nursing and Midwifery, Tabriz University of Medical Sciences, Tabriz, Iran

**Keywords:** Childbirth experience, Questionnaire, Psychometric, QACE, Farsi

## Abstract

**Background:**

Given the importance of the childbirth experience, its effects on women’s life and society, and the need for its assessment by accurate instruments, this study aimed to determine the psychometric properties of the Questionnaire for Assessing the Childbirth Experience (QACE) in an Iranian women population.

**Methods:**

The validity of the Farsi edition of the questionnaire was assessed using the opinions of eight experts. Its construct validity was assessed by studying 530 mothers, at 1–4-month postpartum, who delivered in health centers of Tabriz, Iran. The exploratory factor analysis (EFA) was performed to identify its factors. Then, the confirmatory factor analysis (CFA) was performed for the structural assessment of the extracted factors. Spearman’s correlation coefficient was used to investigate the correlation between factors. Cronbach’s alpha and intraclass correlation coefficient (ICC) were used to obtain the internal consistency and test-retest reliability.

**Results:**

In total, four factors were extracted from the EFA: “relationship with staff” (4 questions), “first moments with the newborn” (3 questions), “feelings at one-month postpartum” (3 questions), and “emotional status” (3 questions). According to the CFA, the model achieved desired fit level (RMSEA < 0.08, GFI, CFI, IFI > 0.90, and x^2^/df < 5.0). Cronbach’s alpha (0.77–0.82) and intraclass correlation coefficient index (0.83–0.98) were desirable for all factors.

**Conclusion:**

The short edition of the QACE, as a standard tool, can be used by future studies to measure the experience of Iranian women.

## Background

Today, the importance of childbirth and its resulting experience is widely recognized. Childbirth may be perceived by women as self-empowerment and a joyful event in their personal lives [1]. It can also be perceived as an unpleasant event with negative impacts, which can persist for years [2]. The traumatic experience of childbirth can disrupt the relationship between mother and newborn, and delay infant development [3]. Postpartum women with a traumatic childbirth experience are more susceptible to postpartum depression and post-traumatic stress disorder than other women [4, 5].

Despite the researchers’ agreement on the importance of the childbirth experience and its short- and long-term effects on the life of the woman and her family, there is little agreement on the conceptual definition of childbirth experience [6]. Nevertheless, most studies have focused only on a few dimensions of the childbirth experience. For example, fear of childbirth, the maternal sense of control over labor and childbirth, maternal and neonatal outcomes, satisfaction with care, perceived professional support, and the severity of pain are regarded as the concept of childbirth experience [6, 7]. Occasionally, the definition of birth trauma may be confused with traumatic or negative childbirth experience [8]. However, the childbirth experience is a complex subjective concept and is not limited to the childbirth outcomes. According to a longitudinal cohort study on 1893 women with a positive experience in Norway, women even with a positive perception of caregivers’ attempts will be satisfied with their childbirth experience, even after having a traumatic experience [9].

In a conceptual analysis, Larkin et al. defined the childbirth as personal life experience comprised of physiological and mental-psychological processes, which are affected by social, environmental, organizational, and policy factors. They characterized childbirth experience by four main characteristics: 1) life event, 2) personal, 3) complex and 4) process [6]. Due to limited definitions of the childbirth experience, there are scant tools to assess this important phenomenon [10]. These tools are also limited to specific childbirth experience dimensions. Instruments, such as “Labor Agentry Scale (LAS)” [11], “Support and Control in Birth (SCIB)” [12], “Wijma Delivery Experience Questionnaire” [13], and “The Pregnancy and Maternity Care Patients’ Experiences Questionnaire” [14] measure only the perceived pain control, perceived control and support, fear of childbirth and satisfaction with caregivers, and maternal perception of her childbirth experience, respectively.

These instruments assess only one dimension or limited dimensions of the childbirth experience. For example, the Wijma Delivery Experience Questionnaire covers only one dimension, i.e. the fear of childbirth, the “Support and Control in Birth” questionnaire measures the control and support dimensions, and the “Labour Agentry Scale” addresses only the control dimension. Due to the broad range and complexity of the concept of the childbirth experience, a comprehensive instrument is needed to measure its different angles. The Questionnaire for Assessing the Childbirth Experience (QACE) was designed by Carquillat et al. in 2017 to assess different dimensions of the childbirth experience concept.

They first adopted the main categories from their previous qualitative studies and other researchers’ studies on key elements of the childbirth experience. The extracted categories included six items: 1) expectations, 2) sensory experience, 3) perceived control, 4) communicating with provider and father, 5) emotions, and 6) first moments with the newborn. The questionnaire has both short and long forms. The long version (25 questions) is introduced as a clinimetric tool; whereas, the short form (13 questions) is introduced as a tool for the overall assessment of a woman’s experience of childbirth. The long-form is introduced as a clinimetric tool because it is not a psychometric scale with theoretically interrelated items. Therefore, as a screening tool, it contains empirically related items. The sort-form QACE (13 questions) has been introduced as a standard tool and its psychometric properties have been confirmed. Its dimensions include the “relationship with staff,” “emotional status,” “first moments with the newborn,” and “feelings one month postpartum” [10]. Given the childbirth experience measurement and the necessity of using a multidimensional tool, the present study aimed to adapt the short version of the QACE among an Iranian women population.

## Methods

### Study design

The present cross-sectional study evaluates the validity and reliability of the Farsi edition of the QACE.

### Study participants and data collection

The participants were selected by three midwifery researchers among women who sought care at health centers (Iran). Inclusion criteria were primiparity, singleton pregnancy, uncomplicated pregnancy and labor, and physical health according to maternal self-report. The exclusion criteria were C-section, history of mental illness according to the maternal self-report, history of a traumatic accident in the family in the past 3 months, hospitalization of the newborn due to postpartum illness, major neonatal malformation(s), and withdrawal during the questionnaire completion stage.

The samples were selected using the cluster sampling method. In this way, 25 out of 87 health centers in Tabriz were randomly selected. All women in Tabriz are covered by one of the health centres and receive care from a general physician or a midwife, who may refer them to a more specialized health centre (university hospitals). These health centres are public and provide women with health services free of charge.

In the selected centers, the list of mothers who were at 1–4-month postpartum was prepared. Eligibility criteria were extracted from electronic records. The researcher made telephone contacts with the eligible women to explain the research objectives and invite them to participate in the study. Those mothers eager to participate were was asked to attend a meeting at health centers. The research questionnaires were completed by the researcher through face-to-face interviews.

### Sample size

According to one rule, the minimum sample size for factor analysis is 300 [15]. However, due to using the cluster sampling design (design effect of at least 1.5) and the probability of 20% data loss, the sample size increased to 530.

### Cross-cultural adaptation procedure

Two midwifery specialists fluent in English translated the original edition from English to Farsi (forward translation). Then, the Farsi edition was back translated into English by an Iranian woman with native-like English proficiency (backward translation). The translator was fluent in both languages and cultures and was blinded to the original version of the questionnaire. After translating the questionnaire, the opinions of eight faculty members in the fields of midwifery, reproductive health, and nursing were obtained qualitatively and quantitatively. Initially, they were asked to examine the questions and their translation into Farsi and modify it, if necessary. In the quantitative phase, they were asked to rate each question for simplicity, relevance, and transparency (Content Validity Index = CVI), and necessity (Content Validity Ratio = CVR). The rating system was scored on a scale of 1 to 4 anchored by “completely,” “relatively,” “not so much,” and “not at all,” respectively. After collecting specialized opinions, the necessary modifications were made.

### Instrument

Data collection instruments were QACE and researcher-made sociodemographic questionnaires. The sociodemographic questionnaire included questions about age, educational attainment, occupation, income, newborn sex, unwanted pregnancy, unwanted sex of baby, etc. The household income level was assessed subjectively and the participants’ responses were given by “relatively sufficient,” “sufficient,” and “insufficient.” The items related to unwanted pregnancy and unwanted sex of baby were also responded by “yes” and “no.” This method for assessing of household income level has been used in other studies [16, 17].

Data related to the childbirth experience was collected using the short QACE (13 questions). The extracted dimensions by Carquillat et al. were “relationship with staff” (4, 5, 6, and 7), “emotional status” (1, 2, and 3), “first moments with the newborn” (8, 9, and 10), and “feelings at one-month postpartum” (11, 12 and 13). Each question is scored from 1 to 4 anchored by “Totally,” “In part,” “Not so much,” and “Not at all,” respectively. Items 2, 12, and 13 were responded inversely. The minimum and maximum achievable scores are, respectively, 1 and 4 - higher scores indicate more negative experience. The Cronbach’s alpha for QACE was at the desired level (0.70 to 0.85) in the study based on Carquillat et al. [10].

### Statistical analysis

Statistical analysis was performed in SPSS25 using a Windows device (IBM Inc., Armonk, NY, USA) and Stata15 (StataCorp, College Station, USA). To describe the participants’ characteristics, the frequency was used for binary variables and the mean (standard deviation) was used for continuous variables. The principal axis factoring (PAF) was used for exploratory analysis and the Oblimin with Kaiser Normalization was applied for rotating factors. In the exploratory analysis, a cut-off point of 0.30 was used to decide which item to keep using the varimax rotation [18]. Spearman’s correlation coefficient was used to investigate the correlation between factors.

After the EFA, the CFA was performed. To evaluate the structure of factors derived from EFA, a CFA model is needed. Based on this model, it should be determined whether the theoretical model presented is validated by data and also if the model’s coefficients are significant. Positive response to these questions confirms the structural validity of the factors. To check the fit of the model, the fit indices are used. The target indices with acceptable values are the root mean square error of approximation (RMSEA) < 0.08, goodness of fit index (GFI), comparative fit index (CFI), and incremental fit index (IFI) > 0.90, and x^2^/df < 5.0 [18].

Cronbach’s alpha and intraclass correlation coefficient (ICC) were used to assess the internal consistency and test-retest reliability. The acceptable values for Cronbach’s alpha coefficient and ICC were above 0.7 and 0.8, respectively [19, 20].

### Ethical consideration

Ethical approval was obtained from the Ethics Committee of Tabriz University of Medical Sciences. Before using the QACE, the required permission was obtained from Dr. Guittier via email. Participants were informed of the purpose of the study and signed a written informed consent form. In the meantime, they were free to leave the study at any time.

## Results

### Characteristics of the participants

A total of 530 mothers completed the questionnaire between January and August 2018. The mean age of the participants was 27 years. The majority of participants (80.3%) had a high school diploma or lower. The characteristics of the participants included in the study are presented in Table 1.

**Table 1:** Baseline characteristics of postpartum mothers (*n* = 530)

Variables	N (%)
Age (years), mean (SD)	27.0 (5.4)
Level of education	
Diploma or below	426 (80.3)
University	104 (19.7)
Occupation status	
Housewife	504 (95.1)
Employee	26 (4.9)
Income level	
Sufficient	57 (10.8)
Relatively sufficient	393 (74.2)
No sufficient	80 (15.1)
Gestational age (weeks)	
Under 37 weeks	65 (12.2)
37 and higher than 37 weeks	465 (87.7)
Unwanted sex of baby	20 (3.8)
Unwanted pregnancy	127 (24.0)

### Content validity

According to the experts, all items had good CVI and CVR values. The range of values obtained for CVI was between 0.91 and 1. Furthermore, all items except 13 items (0.87) achieved the maximum achievable CVR of 1 (Table 2).

**Table 2:** The content validity for Questionnaire for Assessing the Childbirth Experience (QACE)

Item	CVI	CVR
	***N*** = 8-expert	
QACE1	1	1
QACE2	1	1
QACE3	0.91	1
QACE4	1	1
QACE5	1	1
QACE6	1	1
QACE7	1	1
QACE8	1	1
QACE9	1	1
QACE10	1	1
QACE11	0.95	1
QACE12	0.91	1
QACE13	0.91	0.87

### Exploratory factor analysis (EFA)

At the EFA, based on the Scree Plot chart, four factors were separated into the present data. According to the explained variance index, the total predictive power of the model was 77.0, 36.3, 19.1, 10.9, and 10.5% of the variance explained by factors 1, 2, 3 and 4, respectively. All items with at least a minimum score of 0.3 were included in the questionnaire, and none of the items were loaded on two factors. The minimum and maximum values of factor loading were, respectively, 0.52 for “I am proud of myself” and 0.97 for “I felt emotionally supported by the staff who took care of me.”

The same factors from the original study of the instrument spontaneously emerged in the new analysis. Items loaded on factors 1, 2, 3, and 4 were focused on the concepts of “relationship with staff,” “first moments with the newborn,” “feelings at one month postpartum,” and “emotional state,” respectively. Except for Factor 1, on which four items were loaded, the other factors included three items. The mean (standard deviation) of all 13 questions was 1.6 (0.4), and the emotional status factor had the highest mean score (2.4) in the range of 1 to 4. Higher scores reflect more negative childbirth experience (Table 3).

**Table 3:** Factor loading for the Questionnaire for Assessing the Childbirth Experience (QACE)^a^ (*n* = 530)

Item	F1: Relationship with staff	F2: First moments with the newborn	F3: Feelings at 1 month postpartum	F4: Emotional status
1. I felt worried				0.693
2. I felt secure				0.957
3. I felt confident				0.791
4. The staff understood and fulfilled my wishes in a satisfactory manner	0.955			
5. I felt emotionally supported by the staff who took care of me	0.970			
6. The staff kept me informed of what was happening	0.931			
7. I felt I could express myself and give my opinion about decisions about me	0.969			
8. I was able to see my baby for the first time in a satisfactory manner		0.936		
9. I held my baby for the first time when I felt like it		0.968		
10. The first moments with my baby corresponded with what I had imagined prior to giving birth		0.908		
11. I am proud of myself			0.526	
12. I feel regret			0.794	
13. I have a feeling of failure			0.866	
**Percent of variance explained**	36.3	19.1	10.9	10.5
	Total = 77.0			
**Mean (Standard Deviation)**^**b**^	1.7 (0.8)	1.3 (0.7)	1.1 (0.3)	2.4 (0.8)
	Total = 1.6 (0.4)			
**Cronbach’s alpha**	0.82	0.77	0.80	0.79
	Total = 0.82			
**Intraclass Correlation Coefficient (95% CI)**	0.93 (0.81 to 0.97)	0.97 (0.93 to 0.99)	0.98 (0.95 to 0.99)	0.96 (0.90 to 0.98)
	Total = 0.83 (0.56 to 0.93)			

^a^ Extraction Method: Principal Axis Factoring. Rotation Method: Oblimin with Kaiser Normalization; ^**b**^Possiable range: 1–4

### Confirmatory factor analyses (CFA)

According to the values of the indices of this questionnaire, the chi-square was < 5, the RMSEA index was < 0.08, and the RMR < 0.1. These indices confirm the validity of this model. Furthermore, the fit of GFI, AGFI, NFI, NNFI, RFI, IFI, and CFI were > 0.9. As a result, this model achieved a desirable fit level, thereby confirming their factor structure (Table 4).

**Table 4:** Confirmatory factor analyses of the Questionnaire for Assessing the Childbirth Experience (QACE) (n = 530)

Fit indices	χ2/df	RMR	GFI	AGFI	NFI	RFI	IFI	NNFI	CFI	RMSEA (95% CI)
Value	2.31	0.01	0.96	0.94	0.97	0.97	0.98	0.98	0.98	0.05 (0.03 to 0.06)

*χ2* chi-square, *df* degrees of freedom, *χ2/df* normed chi-square, *RMR* Root Mean R, *GFI* Goodness of Fit Index, *AGFI* Adjusted Goodness of Fit Index, *RMSEA* Root Mean Square Error of Approximation, *NFI* Normed Fit Index, *RFI* Relative Fit Index, *IFI* Incremental Fit Index, *NNFI* Non-Normed Fit Index, *CFI* Comparative Fit Index

### Correlation between item-factor and factor-factor

**Fig. 1:**
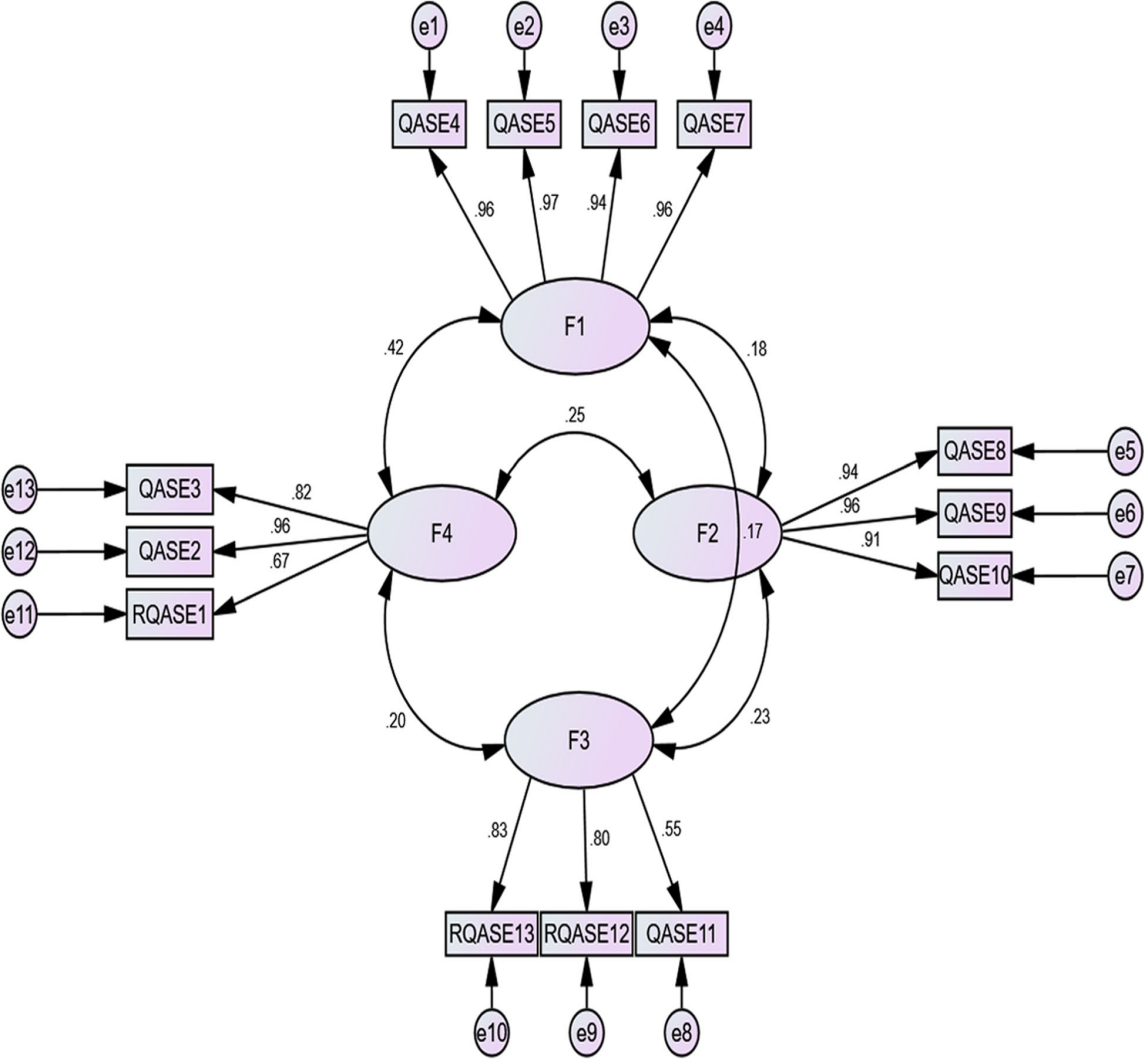
Path diagram with standard coefficients of the Questionnaire for Assessing the Childbirth Experience (QACE)

The results from Table 5 and Fig. 1 show the correlation between each item with its factor and the factors with each other. Although all correlations between factors were statistically significant (*p* < 0.001), these correlations were at either poor or moderate levels. All items had significant (p < 0.001) and moderate to strong correlations (ranging from 0.55 to 0.97) with their factors.

**Table 5:** Pearson correlation coefficient between the four factors of the Assessing the Childbirth Experience (QACE) (*n* = 530)

Factor	Emotional status	First moments with the newborn	Feelings at 1 month postpartum
Relationship with staff	0.17^*^	0.17^*^	0.42^*^
Emotional status	0.23^*^		0.25^*^
Feelings at 1 month postpartum			0.20^*^

^*^ *P* < 0.001

### Discriminant validity

The mean total scores of QACE (p < 0.001), “relationship with staff” (p < 0.001), and “feelings at one month postpartum” (*p* = 0.002) were significantly lower in women with satisfactory marital life than their peers with unsatisfactory marital life. The mean total scores of QACE (*p* = 0.033), “first moments with the newborn” (*p* = 0.017), and “emotional status” (*p* = 0.036) were significantly lower in women with sufficient income than their peers with insufficient or relatively sufficient income (Table 6).

**Table 6:** Discriminante validity for the Assessing the Childbirth Experience (QACE) and it’s four factors (*n* = 530)

Variables	Relationship with staff	First moments with the newborn	Feelings at 1 month postpartum	Emotional status	Total
**Marital satisfaction, mean (SD)**					
Ye	1.5 (0.8)	1.2 (0.7)	1.0 (0.2)	2.3 (0.9)	1.5 (0.4)
No	1.9 (0.6)	1.3 (0.7)	1.1 (0.4)	2.4 (0.6)	1.7 (0.4)
*P*-value^a^	< 0.001	0.497	0.002	0.228	< 0.001
**Income level, mean (SD)**					
Sufficient	1.5 (0.8)	1.2 (0.7)	1.0 (0.2)	2.1 (0.8)	1.5 (0.4)
Relatively sufficient	1.7 (0.7)	1.2 (0.7)	1.1 (0.3)	2.4 (0.7)	1.6 (0.4)
Insufficient	1.7 (0.9)	1.5 (0.9)	1.1 (0.5)	2.5 (0.9)	1.7 (0.6)
*P*-value^b^	0.201	0.017	0.100	0.036	0.033

^a^Independent T-test ^b^one-way ANOVA

### Intraclass correlation index and reliability

The intraclass correlation coefficients were 0.83, 0.93, 0.97, 0.98, and 0.96 for the total questionnaire and factors related to the “relationship with staff,” “first moments with the newborn,” “feelings at one month postpartum,” and “emotional state,” respectively. Therefore, in terms of the intraclass correlation index, the whole questionnaire and its factors obtained good values. Cronbach’s alpha was 0.82, 0.82, 0.77, 0.80, and 0.79 for the total questionnaire, “relationship with staff,” “first moments with the newborn,” “feelings at one month postpartum,” and “emotional state,” respectively (Table 3).

## Discussion

The assessment of the psychometric properties of the Farsi edition of QACE among Iranian women showed that it is a valid and reliable questionnaire for measuring the childbirth experience. According to the results of the exploratory analysis, the number of factors extracted from the Farsi edition was the same as the English version [10]. Similar to the English edition, there were four factors in the Farsi edition. Furthermore, the items loaded on each factor were similar to the English edition. In this study, four factors (i.e., the “relationship with staff,” “first moments with the newborn,” “feelings at one month postpartum,” and “emotional status”) were extracted. The factor loading for all questionnaire items was > 0.3. Besides, CVI and CVR values were in a good range. Therefore, none of the items were removed.

In the Farsi edition, Cronbach’s alpha for the “relationship with staff”, “first moments with the newborn”, “feelings at one month postpartum”, and “emotional status” was 0.82, 0.77, 0.80 and 0.79, respectively. Consistent with the Farsi edition, these values were, respectively, 0.81, 0.84, 0.73, and 0.70 in the English edition [10].

Although the correlations between the instrument’s factors were statistically significant, they were at either a poor or moderate level. Furthermore, in the study of Carquillat et al. [10], these factors had a weak-to-moderate correlation.

The minimum and maximum values of factor loading were, respectively, 0.52 for “I am proud of myself” and 0.97 for “I felt emotionally supported by the staff who took care of me.” In Carquillat et al.’s study [10], the minimum and maximum values of factor loading were, respectively, 0.62 for “I feel regret” and 0.89 for “I held my baby for the first time when I felt like it.” The observed differences in the correlation between the factors and factor loading can be due to the difference in the context of the present study and that of the Carquillat et al.’s study. The maternal care services in Iran differ from those in Swiss and France. For example, in Carquillat et al.’s study, 18.9% of the primiparous women underwent instrumental vaginal delivery; whereas, this technique is rarely used in Iran [21] and none of the participants in the present study received it.

In the present study, the total scores of the childbirth experience, “relationship with staff,” and “feelings at one month postpartum” in women who were satisfied with their marital life were significantly better than those who were not satisfied with their marital life. Consistent with the presents a study, Ghanbari-Homayi et al. [21] also found a statistically significant relationship between marital satisfaction and the childbirth experience. A woman who is satisfied with her husband feels more prepared to give birth. Besides, satisfaction with the husband reduces childbirth-related anxiety and fear [22, 23]. In contrast, a low level of satisfaction with the husband can increase the childbirth-related fear, thereby causing a negative experience of childbirth [24]. Given the high importance of having satisfaction with the husband in acquiring a positive experience of childbirth, the caregivers should try to facilitate involvement and support of the husband throughout pregnancy.

Besides, the total score of factors including the “first moments with the newborn” and “emotional status” in women with sufficient income level was significantly better than those with insufficient or relatively insufficient income. Ghanbari-Homayi et al. [21] reported a statistically significant relationship between income and childbirth experience. Thus, women with inadequate income reported more negative childbirth experience. This finding is consistent with the results of the present study. Women with lower income are less involved with childbirth preparation classes and experience more intense pain during labour, leading to a more negative childbirth experience [25]. Identification of the causes of a positive or negative experience of childbirth contributes to improved services and resources offered by caregivers [26].

The QACE, as a newly developed tool, has not been adapted to other cultures. Therefore, it was not possible to compare the psychometric properties of this tool with other studies. The inclusion of a relatively large number of women in the postpartum period (530 participants) is one of the strengths of this study. The completion of the questionnaire at least four-week postpartum may be another strength of the study. This is because the assessment of satisfaction with the childbirth experience may be influenced by the time of completing the questionnaire, which may not lead to accurate answers [27]. The participants were randomly selected from the women visiting the health centres of Tabriz and thus the findings are generalizable to this group of women. Exclusion of women undergoing C-section was among the research limitations. Therefore, the Farsi edition of this questionnaire cannot be applied to this group of women. The method of assessing income subjectively has not been validated against objective measures and this may be limitation of the study.

## Conclusion

The results of this study showed that the Farsi edition of the QACE is a valid and reliable instrument for the assessment of the postpartum experience in Iranian women. This instrument is recommended in descriptive studies to identify women with the negative childbirth experience, and in interventional studies to assess interventions to improve the childbirth experience and to assess the quality of clinical care. Future studies are recommended to use this questionnaire to predict some maternal-fetal outcomes, such as maternal-fetal attachment, neonatal development, and postpartum psychological problems, caused by negative childbirth experience.

## Data Availability

The datasets used and analysed during the current study are available from the corresponding author on reasonable request.

## Abbreviations

QACE: Questionnaire for assessing the childbirth experience
EFA: Exploratory factor analysis
CFA: Confirmatory factor analysis
ICC: Intra correlation coefficient
CVR: Content validity ratio
CVI: Content validity index
SCIB: Support and control in birth
LAS: Labour agentry scale
PAF: Principal axis factoring
KMO: Kaiser-Meyer-Olkin
SRMSEA: Standardized root mean square error of approximation
CFI: Comparative fit index
TLI: Tucker- Lewis Index
RMR: Root Mean R
GFI: Goodness of fit index
AGFI: Adjusted goodness of fit index
RMSEA: Root mean square error of approximation
NFI: Normed fit index
RFI: Relative fit index
IFI: Incremental fit index
NNFI: Non-normed fit index
